# Environmental Health Literacy of Brazilian Indigenous People

**DOI:** 10.3390/ijerph22040625

**Published:** 2025-04-17

**Authors:** Bernardo Oliveira Buta, Wauana Sheeva Costa Silva Manchineri, Matheus Britto Froner, Maria Berta Ecija, Debora Helena Rosa Cardoso, Benjamin Miranda Tabak

**Affiliations:** 1 School of Public Policy and Government, Getulio Vargas Foundation (FGV-EPPG), SGAN 602 Módulos A,B,C, Asa Norte, Brasilia 70830-020, Distrito Federal, Brazildebora.cardoso@fgv.edu.br (D.H.R.C.); benjamin.tabak@fgv.br (B.M.T.); 2 Wolfson Institute of Population Health, Queen Mary University of London, 58 Turner Street, Yvonne Carter Building, London E1 2AB, UK

**Keywords:** health literacy, environmental health literacy, public health, indigenous people, amazon, climate change

## Abstract

Environmental health literacy (EHL) is essential for individuals to protect themselves from environmental health risks. Indigenous populations are particularly vulnerable to these risks, given the historical threats they have suffered from the advance of agricultural frontiers and impacts of deforestation, mining, and extreme weather events. This study investigates the dimensions of EHL among indigenous communities in Brazil, considering the scarcity of research in this field. Using a scale adapted to measure EHL in topics such as air, water, and food, it was possible to access the EHL levels of a sample of different Brazilian indigenous ethnic groups. Statistical analysis included descriptive methods and the Wilcoxon and Kruskal–Wallis tests. The results revealed significant variations in EHL levels, influenced by factors such as gender, place of residence, age, education, access to health services, and potable water. In addition, the presence of traditional actors, such as midwives, was identified as an important factor in the transmission of health knowledge. The research highlights the need for public policies that respect the cultural specificities of indigenous communities and promote self-care and environmental preservation, contributing to the development of culturally sensitive public health strategies.

## 1. Introduction

Environmental health literacy (EHL) refers to the understanding of the connection between environmental exposures to which individuals are subject and their risks to health [1]. This construct is built on Bloom’s taxonomy, involving knowledge, skills, and attitudes that influence how these elements can be used to make informed choices, reduce health risks, improve quality of life, and protect the population [1,2,3].

Studies on EHL are relatively recent, but they are growing and becoming more relevant, reflecting the greater frequency of the impact of environmental events on human health. Collective changes aimed at mitigating environmental risks initially encompass individual knowledge and awareness of such risks, as well as the development of self-care skills and decisions [4]. This becomes more difficult when it comes to vulnerable populations with higher levels of environmental exposure.

There is growing evidence of climate change and its impacts on indigenous peoples and local communities across the planet. These changes affect their traditional way of life, which has an intimate and intense relationship with the environment. The impacts are diverse, covering the availability of drinking water, air quality, soil humidity, an increase in pests, the abundance of wild and cultivated plants, and the abundance of terrestrial and aquatic animals [5].

Indigenous territories have historically been threatened by the expansion of agribusiness, intensifying the vulnerability of indigenous populations to the impacts of these activities, such as deforestation, biodiversity loss, and disruption in their way of life [6,7]. Indigenous peoples play an essential role in preserving biodiversity. Their worldview, associated with their way of life, promotes the sustainable use of natural resources, making the protection and preservation of their territories an important instrument for environmental conservation [6,8,9].

Brazil has approximately 1.7 million indigenous people. Most of this population (51.2%) is concentrated in the Legal Amazon, according to data from the 2022 Census [10]. Nevertheless, there is still little production on indigenous populations in the literature on EHL, which is an important gap in knowledge. Despite the environmental risks to the health of indigenous communities, the literature on EHL has not yet focused on this population. This can be seen from a search in the Scopus, WOS, and PubMed databases for the terms “environmental health literacy”, “indigenous”, “aborigines”, “ethnic groups”, “first nations”, “native”, “natives” or “traditional”, which returned only five articles. To help fill this gap, the aim of this study was to explore the dimensions of EHL among indigenous people in Brazil. To this end, we used the scale developed by Lichtveld et al. [2] with adaptations.

It is important to point out that not many studies have been conducted on the socio-environmental determinants of the health of indigenous communities, although this perspective is especially valid for indigenous communities, who, despite being important players in the defense of the environment, suffer significant impacts from extreme environmental events [5]. These socio-environmental determinants include but are not limited to factors such as infrastructure, income, access to education and health services, living conditions, and risk behaviors [11]. Climate change itself can also be seen as a social determinant of health [11,12].

EHL levels tend to vary according to factors such as income [13], education [13,14,15,16,17], age [14,16], gender [14,16,18], living situation (live alone or with family) [15], marital status [15,16], rented or owned home [14], place of residence [15,17], political ideology, and belief in science [14].

These aspects are highlighted as important indicators of behaviors and lifestyle because they are also understood and classified as some of the most relevant social determinants of health (SDHs). Those are non-medical factors, such as the conditions that people live, grow, age, and die [19], that play a significant role in populations’ basic access to resources that influence their health outcomes and quality of life.

## 2. Method

### 2.1. Data Collection

This exploratory research was conducted by applying EHL scales [2] to Brazilian indigenous people. These EHL scales have been tested in various samples globally [16,20,21] and have undergone a cross-cultural validation process in Brazil, although the results have not yet been published. The survey instrument is available in the Supplementary Material.

These scales assess EHL in general, as well as specifically on the topics of food, air, and water. Each scale encompasses three dimensions: knowledge, which pertains to facts and information about environmental health acquired through experience or education; attitudes, which refer to established ways of thinking or feeling towards environmental health; and behaviors, which include the actions individuals take in response to environmental health concerns [2]. Responses to the questionnaire were collected using a five-point Likert scale, which measures either agreement (ranging from strongly agree to strongly disagree) or frequency (ranging from always to never). The calculation of individual scores was based on the average of the responses from each participant.

In addition to the items related to EHL, the data collection instrument included questions for the socioeconomic characterization of the respondents and the infrastructure of their communities. These questions covered various issues, such as the availability of schools and the languages spoken there, the presence of health agents, energy supply, and the availability of Internet access and telephone communication services.

Data collection occurred between July and December 2023 and was conducted in two stages. The first stage involved a pilot application of the questionnaire to 41 Indigenous participants, during which researchers were present to note any uncertainties or comprehension difficulties encountered. Following this phase, it was necessary to remove items that were not culturally appropriate and to revise the wording of items that were semantically unclear. The second stage involved personally administering the revised questionnaire to an additional 107 participants. After excluding cases with significant missing data, which suggested participant abandonment, 102 observations remained.

### 2.2. Participants

The survey was administered to Indigenous people from various ethnicities, who were conveniently selected from different Brazilian states, mainly including all states within the Brazilian Amazon. This region is home to 51% of the Brazilian Indigenous population, approximately 870,000 people, as reported by the most recent national census conducted in 2022. This population is notably difficult to access. Therefore, interactions with participants occurred in Brasília, during events sponsored by the Coordination of Indigenous Organizations of the Brazilian Amazon (COIAB). The survey also included a small group of Indigenous residents of Brasília and Indigenous students from the University of Brasília.

The sample comprised 52.9% females. Furthermore, 38.2% were between 18 and 29 years old, and 41.2% did not have access to higher education, with 21.6% not even completing secondary education. Additionally, 62.7% of the respondents lived in indigenous villages. Regarding social roles, 28.4% were political leaders within their communities, 13.7% were teachers, 5.9% were agents of health or sanitation, and 3.9% were religious leaders. Table 1 presents a profile of the sample.

### 2.3. Data Analysis

Data analysis was performed using non-parametric association tests, since the sample did not come from a normal distribution [22]. We used the Wilcoxon two-sample statistic for comparing EHL scores between two groups, for example, males and females, or indigenous people living in villages and indigenous people living in the city; a Kruskal–Wallis rank-sum test for comparing EHL scores between three or more groups, for example, groups of people of different ages, or groups with different education levels; and Dunn’s test, after the Kruskal–Wallis test, for multiple pairwise comparisons.

We also used descriptive statistics to estimate the level of EHL of the sample across the different themes: air, water, food, and general. Lichtveld et al. [2] did not specify methods for computing EHL levels or criteria for assessing levels of EHL. Consequently, we decided to compute the EHL by taking the means of the responses, which were spread across a five-point Likert scale. We reversed the scoring for one item from the general scale and for four items from the air scale.

To determine the EHL levels of respondents and ensure uniform classification within this study, we defined the following categories: EHL was considered inadequate if below 2.99; problematic if it ranged between 3.00 and 3.99; and sufficient if it exceeded 4.00. Consequently, individuals with an EHL greater than 4 were equipped with the essential knowledge, attitudes, and behaviors required to prevent or reduce environmental health risks.

## 3. Results

Table 2 presents a description of EHL scores. Lower levels of EHL were observed in the air topic, and the highest levels were in relation to the food topic. Values for air EHL and water EHL were considered problematic, whereas general EHL and food EHL values were considered sufficient. A relatively homogeneous variation was also observed for all EHL topics but with the largest portion of individuals having scores below average.

The data shown in Table 3 indicated that, when considering gender characteristics, men showed a higher level of EHL on the air scale compared to women. However, for the food, water, and general scales, there were no significant differences between the genders.

The analysis by age group revealed that individuals aged 40 to 49 had the highest level of general EHL compared to other age groups. On the other hand, the 30–39 age group stood out for its lower literacy in relation to food. It was not possible to say whether there were significant differences for the other age groups.

Regarding education, individuals with completed higher education (including those with incomplete or complete higher education and postgraduate degrees) exhibited a higher level of EHL on the air scale.

Regarding the place of residence, individuals who lived in cities had a higher level of EHL in relation to air than indigenous people who lived in villages. In addition, the analysis of the social role of individuals did not allow us to say whether there were significant variations in EHL levels between different social roles, such as political leaders, religious leaders, health workers, and teachers.

Table 4 refers to the infrastructure of indigenous communities, including energy supply, water supply, availability of means of communication, existence of a local school, and the language in which subjects are taught. Initially, the existence of physical structures for certain services in the community, such as a health center, school, and buildings for community meetings, was not associated with the respondents’ level of EHL.

Regarding the association between EHL and energy sources, it was not possible to say whether there were significant differences in EHL levels for any of the scales. As for the association between EHL and forms of water distribution, communities with a general distribution network had higher levels of EHL on both the water and general scales. It could also be seen that communities that had any form of water distribution, whether through a piped water network, cisterns, or community wells, had higher levels of EHL in relation to water than communities that did not have any form of water distribution.

Concerning the availability of communication resources and the availability of means of communication, respondents from communities that had working public telephones had a higher EHL level on the food and water scales when compared to other communities that had access to the Internet, satellite dishes, or radios.

Looking at the availability of local schools and the provision of subjects taught in Portuguese, no significant associations were observed between these variables and levels of EHL. However, it should be noted that respondents from communities where the children studied in the city had higher levels of EHL on the air and food scales compared to those from communities where the children studied in a neighboring community.

Table 5 below shows the results of the association between the levels of EHL and the characteristics of the communities in terms of health services, including the availability of indigenous health or sanitation agents, the frequency of visits by health teams to the community, and the existence of social actors responsible for maintaining traditional knowledge.

When we considered the availability of health and sanitation agents, whether indigenous or non-indigenous, individuals from communities that did not have these professionals presented a lower level of water EHL compared to those from communities where there were frequent visits from health or sanitation agents. In other words, the presence of health teams in the community, even if infrequent, was associated with higher levels of EHL about water quality.

Regarding the existence of social actors responsible for maintaining traditional knowledge, such as shamans, midwives, and benzedeiras, the presence of traditional indigenous midwives was associated with a higher level of general EHL.

## 4. Discussion

The results showed significant variations in levels of EHL. It was also observed that some social factors could act as determinants of the level of health literacy. These social factors can be understood as socioeconomic status, community networks, and cultural aspects that play a significant role in how individuals will perceive the quality of services and resources available to them. In the context of this article, we looked at these determinants through the lenses of the environmental implication to the quality of life, health, and lifestyle. These are broken down in the discussion by looking at the following determinants: constitutional factors, mainly age, gender, and ethnicity, followed by aspects related to community networks and accessibility to health resources, such as education, infrastructure (energy supply, water and sanitation), and housing [23].

Men presented higher levels of air-related EHL than women. The literature has shown that men and women behave differently when faced with environmental health risks. For instance, in certain contexts, men tend to adopt positive behaviors towards the domestic environment, and women tend to show positive attitudes towards food consumption [18], as well as protective behaviors in relation to contagious diseases [14].

Regarding age, on the one hand, individuals aged between 40 and 49 had higher levels of general EHL compared to other age groups. On the other hand, individuals in the 30–39 age group had lower levels of food EHL than those in other age groups. These results may have been due to a sampling bias. In this sense, further studies are needed to explore this issue further.

Education was another factor associated with EHL. Individuals with higher education attainment stood out with higher levels of air EHL. In that case, there is evidence in the literature indicating a positive relationship between education and EHL [13,14,15,16].

Individuals living in cities had a higher level of EHL in the air dimension than individuals living in villages. In fact, there is evidence in the literature of higher levels of EHL in urban areas, suggesting that geographical location influences the level of EHL [17]. In our case, it is important to note that air quality tended to be worse in big cities than in forest villages. In this sense, people who suffered problems related to outdoor air pollution tended to know more about the health risks of this environmental factor and act to mitigate that risk. In addition, within indigenous territories, one of the factors that can influence the perception of air pollution is fires, so future studies focusing on this topic should be carried out.

As for the presence of health services in the community, there was no association between this factor and EHL. However, the absence of health services was significantly associated with low levels of EHL. This indicates that the presence of health and sanitation professionals and teams, even if through infrequent visits, is important for the population to gain knowledge about environmental health risks and act to mitigate them. In fact, there is evidence in the literature of the value of community health workers in raising awareness about environmental health risks [4].

There was no association between EHL and energy distribution sources, nor were there any associations with various means of communication. This may indicate that health information tends to be obtained through personal interaction, rather than the forms traditionally used by Western society, such as the Internet and mass media. Further studies would be necessary to delve deeper into this point. Adaptations of the eHEALS instrument [24] could be one way of obtaining data in this regard.

The results also indicated that communities with some form of water distribution, such as piped water, cisterns, or community wells, had higher levels of EHL on the water scale than those without this infrastructure. This suggests a relationship between the availability of treated water and awareness of the health risks of water use. In any case, it is not possible to specify the direction of causality of these relationships. For example, community-level results of EHL interventions include increased community involvement in environmental issues, culminating in investments such as the provision of alternative means of water supply [4,25].

No associations were found between EHL and the presence of schools in the community or the language in which the subjects were offered. However, it was observed that respondents from communities where students studied in the city had significantly higher EHL in the air and food dimensions. It is possible that these communities were urban or located closer to cities. Proximity to an urban setting may also mean greater access to resources, which tends to have an influence on the educational service.

It is important to consider the Brazilian National Common Core Curriculum, which defines the competencies expected for elementary school (children aged 6 to 14). It is expected that, during this period of development, students acquire knowledge about health, basic sanitation, air quality, and nutritional conditions. Although educational legislation guarantees indigenous peoples the same right to a differentiated education, in fact, this right has not been respected. Indigenous schools face precarious infrastructures and pedagogical practices, resulting in poor-quality education for these communities [26].

Finally, the existence of social actors responsible for maintaining traditional knowledge, such as indigenous midwives, was significant. This suggests that the presence of indigenous midwives contributes to the transmission of fundamental knowledge related to health and the environment, promoting greater understanding and care for the environment.

## 5. Conclusions

With the aim of exploring the EHL dimensions of indigenous people in Brazil, this study identified possible determinants of EHL in a sample of the Brazilian indigenous population. However, there are still avenues of research to be explored in greater detail so that interventions and public policies can be devised to promote greater awareness of the environmental health risks faced by such a diverse, vulnerable, and little-studied section of the population group.

The disparities found in the results reinforce the need for public policies that consider the specificity of each group, especially in terms of gender, age, education, ethnicity, living conditions, and life systems. To an extent, these findings showcase that some lifestyle and health outcomes are not a matter of individual choice. They are predetermined by a series of socioeconomic and environmental determinants that people live under. Thus, in the formulation of policies, we need a more holistic approach that encompasses socioeconomic disparities and community networks as significant indicators of potential vulnerabilities, in accessibility to health-related resources [23].

Environmental health education strategies that take these factors into consideration may be more effective in promoting self-care and environmental protection among indigenous communities, since they will seek to develop self-awareness, knowledge, skills, and changes in community behavior. The results of this study also highlight the importance of considering both the need for formal health and education services and the presence and strengthening of traditional actors, such as traditional midwives, in the promotion of EHL in different dimensions, especially those linked to the management of vital resources such as water and food.

One of the main contributions of this study is to begin mapping the understanding of EHL among indigenous peoples, highlighting the importance of traditional factors, such as the presence of midwives, the transmission of fundamental knowledge related to health and the environment. This approach reinforces the importance of local knowledge and cultural practices in promoting health and sustainability in these communities. In addition, the study opens up a new level of research, contributing to the formulation of public policies that are more in line with the indigenous reality.

However, to make progress in the search for effective solutions, it would be advisable to broaden the scope of this study to include a wider representation of the various indigenous groups. The current study had certain limitations in that it focused on a political elite of indigenous representatives, while the cultural and linguistic diversity is vast.

In addition, it is considered advantageous to adopt diversified research models, which include both comprehensive studies and specific interventions. It is also suggested to incorporate analyses aimed at understanding the dimensions of the direct impacts of climate change, deforestation, fires, and mining on indigenous territories, since these phenomena represent significant threats to the integrity, survival, and health of these peoples.

This study is not free of limitations. As the data collection strategy covered indigenous people at COIAB events, there is a selection bias, as the respondents were part of a political elite. This may lead to a limitation of the research, which may not represent the population as a whole. However, it should be noted that this elite had a strategic vision and in-depth knowledge of their communities and was elected by their people as indigenous leaders to represent them in strategic political spaces. In addition, different studies may adopt varying criteria for interpreting EHL scores. In this study, the thresholds were defined based on the interpretation of the five-point Likert scale, which may influence comparability with other studies.

## Figures and Tables

**Table 1:** Descriptive statistics of the sample.

Feature	%
**Gender**	
Male	47.1
Female	52.9
**Age**	
18–29	38.2
30–39	26.5
40–49	27.5
50–59	6.9
60+	1.0
**Education**	
No formal education	2.0
Primary education	10.8
Incomplete secondary education	8.8
Complete secondary education	16.7
Complete technical secondary education	2.9
Incomplete higher education	30.4
Complete higher education	21.6
Completed postgraduate degree	6.9
**Living situation**	
Indigenous villages	62.7
Cities	37.3
**Social Roles**	
Political leaders	28.4
Religious leaders	3.9
Agents of health or sanitation	5.9
Teachers	13.7
Other	48.1

**Table 2:** Descriptive statistics of EHL scores.

	N	Mean	Median	sd	Min	Max	Range	Skewness	Kurtosis
**Air EHL**	102	3.50	3.50	0.54	1.5	4.83	3.33	−0.31	3.87
**Food EHL**	102	4.28	4.17	0.5	3.0	5.00	2.00	−0.21	2.47
**Water EHL**	102	3.80	3.82	0.49	2.5	4.82	2.32	−0.36	2.87
**General EHL**	102	4.00	4.12	0.52	2.5	4.87	2.37	−0.52	2.79

**Table 3:** Average EHL score by feature.

Feature	n	Air	Food	Water	General
**Gender**					
Male	48	3.61 *	4.29	3.72	3.94
Female	54	3.40	4.28	3.88	4.06
**Age**					
18–29	39	3.5	4.2	3.78	3.83
30–39	27	3.45	4.09 ***	3.70	3.99
40–49	28	3.58	4.48 ***	3.92	4.22 ***
50+	8	3.33	4.56	3.87	4.12
**Education**					
Low educational attainment	22	3.35	4.38	3.85	4.08
Medium educational attainment	20	3.35	4.31	3.73	3.95
High educational attainment	60	3.61 *	4.23	3.81	3.99
**Living situation**					
Indigenous villages	64	3.41	4.29	3.79	4.01
Cities	38	3.66 **	4.27	3.83	3.99
**Social roles**					
Political leaders	29	3.44	4.38	3.82	4.02
Religious leaders	4	3	4.08	4	4.44
Agents of health or sanitation	6	3.39	4.31	3.48	4.06
Teachers	14	3.21	4.30	3.82	4.16
Other	53	3.58	4.23	3.84	3.94

Note: * *p* < 0.05, ** *p* < 0.01, *** *p* < 0.001. Statistical comparisons are made across columns (differences in the same EHL domain between different groups).

**Table 4:** Average EHL score by feature.

Feature	n	Air	Food	Water	General
**The community has**					
Health station or similar	55	3.52	4.29	3.82	4.01
No	47	3.47	4.27	3.79	3.99
School or place to hold classes	78	3.48	4.25	3.79	3.96
No	24	3.56	4.37	3.87	4.15
Place for parties, meetings, or other community activities	64	3.51	4.24	3.81	3.96
No	38	3.48	4.36	3.80	4.09
**The energy supply comes from**					
Public supply network	52	3.51	4.30	3.85	4.02
Other	50	3.49	4.26	3.76	3.99
Electric generator for community supply	24	3.51	4.14	3.68	3.84
Other	78	3.49	4.32	3.84	4.05
Community solar panel	21	3.40	4.41	3.87	4.02
Other	81	3.52	4.25	3.79	4.00
Any of the previously mentioned power supply	88	3.47	4.30	3.82	4.00
No	14	3.64	4.17	3.70	4.04
**The community has**					
General water distribution network	31	3.49	4.38	3.96 **	4.23 ***
No	71	3.5	4.23	3.73	3.90
Water tank or water cistern	41	3.54	4.33	3.88	4.04
No	61	3.47	4.25	3.75	3.98
Community well	34	3.48	4.26	3.86	3.96
No	68	3.51	4.29	3.78	4.03
Any of the previously mentioned water distribution	82	3.47	4.30	3.88 **	4.05
No	20	3.61	4.19	3.52	3.83
**The community has**					
Public telephone in operation	18	3.54	4.51 **	3.98 *	4.16
No	84	3.49	4.23	3.77	3.97
Internet access that can be used by the entire community	60	3.47	4.31	3.81	4.01
No	42	3.54	4.24	3.79	3.99
Satellite dish in operation for the entire community	18	3.49	4.43	3.85	4.01
No	84	3.50	4.25	3.79	4.00
Cell phone network	28	3.57	4.30	3.84	4.02
No	74	3.47	4.27	3.79	4.00
Radio communication system	24	3.44	4.24	3.82	3.93
No	78	3.51	4.29	3.80	4.03
Any of the previously mentioned media	90	3.50	4.30	3.81	4.01
No	12	3.44	4.12	3.78	3.93
**Is there a school on site?**					
Yes	89	3.49	4.29	3.83	4.00
No	13	3.53	4.24	3.65	4.00
**At school, are subjects taught in Portuguese?**					
Yes	77	3.50	4.30	3.87	4.03
No	9	3.39	4.17	3.52	3.80
Not applicable	16	3.53	4.24	3.65	4.00
**Where do community children study?**					
In a neighboring village	6	3.17	3.94	3.59	3.94
In the town	7	3.83 **	4.5 *	3.71	4.05
Other	89	3.49	4.29	3.83	4.00

Note: * *p* < 0.05, ** *p* < 0.01, *** *p* < 0.001.

**Table 5:** Average EHL score by feature.

Feature	Air	Food	Water	General	
**The community is served by:**					
Non-indigenous health or sanitation agent	20	3.5	4.34	3.89	4.13
Indigenous health or sanitation agent	76	3.49	4.25	3.80	3.98
Not served	3	3.72	4.67	3.30 *	3.71
**How often does the health team visit the community?**					
There is a team in the community	23	3.49	4.26	3.79	3.88
Weekly	5	3.57	4.13	3.85	3.72
Fortnightly	17	3.44	4.36	3.95	4.26
More than once a month	7	3.52	4.29	3.71	4.04
Once a month	42	3.49	4.28	3.86	4.05
Never	2	3.58	3.67	2.77 **	3.5
Does not apply	6	3.72	4.67	3.30	3.71
**The community has**					
Pajé/shaman	70	3.45	4.28	3.79	4.02
No	32	3.60	4.28	3.84	3.96
Midwife	58	3.56	4.30	3.82	4.09 **
No	44	3.41	4.25	3.78	3.87
Prayer/blesser	54	3.55	4.27	3.77	4.00
No	48	3.43	4.30	3.85	4.00
Any of the previously mentioned social players	92	3.49	4.27	3.81	4.00
No	10	3.57	4.37	3.75	4.07

Note: * *p* < 0.05, ** *p* < 0.01.

## Data Availability

The dataset can be obtained from the corresponding author upon reasonable request.

## Supplementary Materials

The following supporting information can be downloaded at: https://www.mdpi.com/article/10.3390/ijerph22040625/s1, Table S1: Original and adapted items from the EHL scale.
