# Supplementary material for: Environmental Health Literacy of Brazilian Indigenous People
Source: Int J Environ Res Public Health. 2025 Apr 17;22(4):625. doi: 10.3390/ijerph22040625 (PMC12026955; doi:10.3390/ijerph22040625)
Supplement: Supplementary file 1 [file ijerph-22-00625-s001.zip › ijerph-3466486-supplementary.pdf]

Table 1: Original and adapted items from the EHL scale

**Original Items**	**Adapted Items**
**Air Scale**	
Storing chemicals like gasoline inside the home is not a problem as long as the container is closed.	Armazenar produtos químicos como gasolina dentro de casa não é um problema, desde que o recipiente esteja fechado.
The air quality in my community is impacted by local industry.	A qualidade do ar em minha comunidade é afetada pela indústria local.
Products that are used to freshen indoor air always improve indoor air quality.	O uso do ar-condicionado melhora a qualidade do ar interno. (Modified for better understanding)
Indoor air pollution is not a problem in my state.	A poluição do ar em ambientes fechados é um problema para minha saúde. (Modified for better understanding)
Air pollution does not affect my or my family's health.	A poluição do ar não afeta a minha saúde ou a de minha família.
I consider the air I breathe in my community to be clean.	Considero que o ar que respiro em minha comunidade é limpo.
I have had my indoor air tested.	Excluded
I use face masks when cleaning my house.	Eu uso máscaras faciais ao limpar minha casa.
I avoid exercising because of pollution.	Excluded
I avoid opening my window due to poor outdoor air quality.	Excluded
**Food Scale**	
Washing hands when making meals helps keep disease from spreading.	Lavar as mãos antes das refeições ajuda a evitar a propagação de doenças.
Cutting a tomato on a cutting board after cutting raw meat without washing the board might lead to cross-contamination and spreading of disease.	Cortar um tomate em uma tábua de corte depois de cortar carne crua sem lavar a tábua pode levar à contaminação cruzada e à disseminação de doenças.
I believe that learning about food safety will benefit my health.	Acredito que aprender sobre segurança alimentar beneficiará minha saúde.
I am willing to attend a food safety training course.	Excluded
I select a place to purchase groceries based on its reputation and cleanliness.	Escolho um local para comprar mantimentos com base em sua reputação e limpeza.
I select a restaurant based on its reputation, cleanliness, and food safety score.	Escolho um restaurante com base em sua reputação, limpeza e pontuação de segurança alimentar.
I am willing to change my food handling behaviors when I learn they are unsafe.	Estou disposto a mudar meus comportamentos de manipulação de alimentos se souber que eles não são seguros.

(Continued)

**Original Items**	**Adapted Items**
I use separate clean utensils to handle raw and fresh items while cooking.	Uso utensílios limpos e separados para manusear itens crus e frescos durante o cozimento.
I use utensils to handle food that is ready to eat.	Uso utensílios para manusear alimentos que estão prontos para comer.
**Water Scale**	
Chlorine is used to kill bacteria in water systems.	O cloro é usado para matar bactérias nos sistemas de água.
Municipal (city) drinking water is processed at a water treatment facility before it is delivered to the public.	A água potável municipal (da cidade) é processada em uma instalação de tratamento de água antes de ser entregue ao público.
The government oversees the quality of the drinking water in cities around the country.	O governo supervisiona a qualidade da água potável nas cidades.
Shampoo and out-of-date medications flushed in the drain can be harmful to our water supply.	Xampu e medicamentos fora da validade jogados no ralo podem ser prejudiciais ao nosso abastecimento de água.
I often worry about safe drinking water.	Eu me preocupo se a água que consumo é potável.
I worry about chemicals in our drinking water.	Preocupo-me com os produtos químicos em nossa água potável.
I worry about the quality of water because of old pipes in our homes.	Preocupo-me com a qualidade da água que uso para o meu consumo.
I only use the dishwasher when I have a full load.	Excluded
I only wash clothes when I have a full load.	Lavo roupas quando tenho roupas o suficiente para encher a máquina de lavar. Em caso de ausência de máquina de lavar roupas, deixe a resposta em branco.
I pay attention to how much time I spend in the shower in an effort to conserve water.	Presto atenção ao tempo que passo no chuveiro para economizar água. Caso não faça uso de chuveiro, deixe a resposta em branco.
I track water usage monthly using my water bill.	Acompanho o consumo de água mensalmente. (Modified for better understanding)
I comply with instructions when a boil water advisory is issued by the city.	Excluded
I turn off the tap water while brushing my teeth.	Desligo a água da torneira quando estou escovando os dentes.
I do not open the tap all the way while washing dishes.	Abro totalmente a torneira ao lavar a louça.
**General Scale**	
Chemicals can be found in carpet, rugs, curtains, and furniture.	Excluded

(Continued)

Original Items	Adapted Items
Second-hand smoking is harmful to health.	O fumo passivo é prejudicial à saúde. (Considere ”fumo passivo” como inalação de fumaça de derivados do tabaco por indivíduos não fumantes).
Cutting a tomato on a cutting board after cutting raw meat without washing the board might lead to cross-contamination and spreading of disease.	Cortar um tomate em uma tábua de corte depois de cortar carne crua sem lavar a tábua pode levar à contaminação cruzada e à disseminação de doenças.
I worry about the chemicals I am exposed to on a daily basis.	Eu me preocupo com os produtos químicos aos quais estou exposto diariamente.
I worry about chemicals because they are always bad for my health.	Excluded
I think pollution is a problem, but there is nothing I can do to fix it.	Eu procuro, com minhas ações, contribuir para resolver o problema da poluição.
I avoid inhaling car exhaust.	Excluded
I avoid inhaling cleaning products.	Excluded
I avoid exposing myself and family members to harmful chemicals.	Excluded

Source: *Lichtveld et al., 2019.*
